# Decrease of 5hmC in gastric cancers is associated with *TET1* silencing due to with DNA methylation and bivalent histone marks at *TET1* CpG island 3′-shore

**DOI:** 10.18632/oncotarget.6069

**Published:** 2015-10-10

**Authors:** Jong-Lyul Park, Hee-Jin Kim, Eun-Hye Seo, Oh-Hyung Kwon, Byungho Lim, Mirang Kim, Seon-Young Kim, Kyu-Sang Song, Gyeong Hoon Kang, Hyun Ja Kim, Bo Youl Choi, Yong Sung Kim

**Affiliations:** ^1^ Epigenome Research Center, Genome Institute, KRIBB, Daejeon, Republic of Korea; ^2^ Department of Functional Genomics, Korea University of Science and Technology, Daejeon, Republic of Korea; ^3^ Department of Pathology, College of Medicine, Chungnam National University, Daejeon, Republic of Korea; ^4^ Department of Pathology, Seoul National University College of Medicine, Seoul, Republic of Korea; ^5^ Departments of Preventive Medicine, College of Medicine, Hanyang University, Seoul, Republic of Korea

**Keywords:** gastric cancer, TET1, DNA methylation, 5-hydroxymethylcytosine, 3′-shore, bivalent mark

## Abstract

Recent evidence has shown that the level of 5-hydroxymethylcytosine (5hmC) in chromosomal DNA is aberrantly decreased in a variety of cancers, but whether this decrease is a cause or a consequence of tumorigenesis is unclear. Here we show that, in gastric cancers, the 5hmC decrease correlates with a decrease in *ten-eleven translocation* 1 (*TET*1) expression, which is strongly associated with metastasis and poor survival in patients with gastric cancer. In gastric cancer cells, *TET*1-targeted siRNA induced a decrease in 5hmC, whereas *TET*1 overexpression induced an increase in 5hmC and reduced cell proliferation, thus correlating decreased 5hmC with gastric carcinogenesis. We also report the epigenetic signatures responsible for regulating *TET*1 transcription. Methyl-CpG Binding Domain Sequencing and Reduced Representation Bisulfite Sequencing identified unique CpG methylation signatures at the CpG island 3′-shore region located 1.3 kb from the transcription start site of *TET*1 in gastric tumor cells but not in normal mucosa. The luciferase activity of constructs with a methylated 3′-shore sequence was greatly decreased compared with that of an unmethylated sequence in transformed gastric cancer cells. In gastric cancer cells, dense CpG methylation in the 3′-shore was strongly associated with *TET*1 silencing and bivalent histone marks. Thus, a decrease in 5hmC may be a cause of gastric tumorigenesis owing to a decrease in *TET*1 expression through DNA methylation coupled with bivalent marks in the 3′-shore of *TET*1.

## INTRODUCTION

DNA methylation is a common genomic modification, and methylation at the 5′-position of cytosine (5mC) is a key component of this epigenetic mark that is essential for silencing of repetitive elements, X-chromosome inactivation, and imprinting in the mammalian genome. De novo methylation at CpG islands (CGIs) is associated with transcriptional silencing of many cancer-related genes [1, 2]. Recent evidence has shown that 5mC can be converted to 5-hydroxymethylcytosine (5hmC) by the ten-eleven translocation (TET) family proteins [3] and that 5hmC may be an intermediate in DNA demethylation processes that result in the conversion of 5mC to cytosine [4]. However, the function and underlying regulatory mechanisms of 5hmC DNA modification have not been fully elucidated, and whether 5hmC serves solely as a precondition for DNA demethylation or has a separate regulatory role in demethylation is unclear [5].

Recent studies have shown that the amount of 5hmC in chromosomal DNA is substantially decreased in many types of cancers and that this decrease is associated with decreased *TET* family expression [6]. In addition, somatic mutations in *isocitrate dehydrogenase* (*IDH*) family genes have been identified in several cancers [7-10]. *IDH*1/2 mutations reduce cellular α-ketoglutarate level and result in accumulation of 2-hydroxyglutarate, which inhibits TET activity, resulting in a decrease in 5hmC [11]. A recent report showed that this decrease in 5hmC is associated with certain clinicopathological features of gastric cancers (GCs) [12]. However, little is known about the possible correlation among the level of 5hmC, *TET* family expression, and *IDH* family mutations in GCs. Furthermore, the mechanism of how *TET* expression is controlled in human cancers remains unsolved.

GC is the second leading cause of cancer-related death worldwide owing to its high prevalence [13,14]. Although the clinical outcome of GC has gradually improved, GC diagnosis is often delayed owing to the absence of early specific symptoms, and consequently many patients have advanced disease at the time of initial diagnosis. Thus, a pressing need exists for useful biomarkers that define the malignant potential of primary gastric tumors (GTs), predict prognosis, and establish new therapeutic and preventive strategies for this disease.

The promoter regions of silenced genes, including those with promoter DNA methylation, contain specific histone modifications that ensure transcriptional inactivation [15]. Additionally, the DNA methylation mark itself can be read by specific proteins that alter chromatin structure [16]. Thus, cross-talk exists between DNA methylation and histone modifications to orchestrate transcriptional silencing. Studies have shown that bivalent domains mark the promoters of genes that will become methylated in adult tumor cells to reinforce transcriptional silencing [17, 18].

Here we reveal that a decrease in 5hmC level is common in gastric tumors, is strongly associated with decreased expression of *TET*1, and is significantly associated with poor survival of patients with GC. Furthermore, we demonstrate that expression of *TET*1 itself is controlled by epigenetic regulation, including DNA methylation coupled with bivalent marks, of CGI 3′-shore regions rather than promoter CGIs. Thus, our data suggest that a decrease in 5hmC level or *TET*1 expression may be a prognostic biomarker for GC and that CpG methylation on 3′-shores may be a target for epigenetic editing to manage patients with GC.

## RESULTS

### Decreased global 5hmC in primary GTs

**Figure 1:**
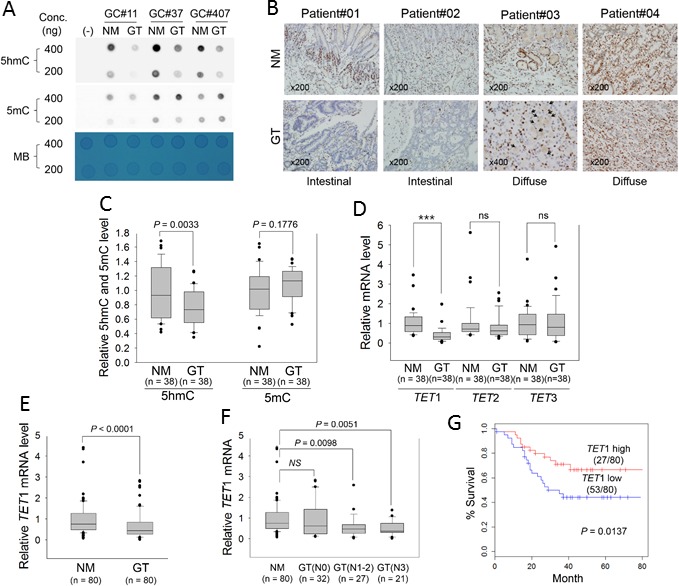
Measurement of global 5hmC and 5mC and expression of *TET* family genes in primary GTs **A.** Dot blot assay for global levels of 5hmC and 5mC in three paired GTs and adjacent NMs with two different genomic DNA concentrations. A PCR product was used as negative control (−). MB, methylene blue staining (to estimate the relative concentration of DNA). **B.** Comparison of the 5hmC signature between NMs (upper) and GTs (lower) in FFPE tissues. All panels show immunostaining results with anti-5hmC. Patient#01 and #02: intestinal-type GCs; patient #03 and #04: diffuse-type GC (×200). Black arrowheads in the bottom panel for Patient#03 indicate the signet ring cells. **C.** ELISA for quantitation of 5hmC and 5mC in 38 paired GTs and adjacent NMs, according to the standard curves shown in Supplementary Figure 1. **D.** Quantitative real-time RT-PCR for *TET* family genes in GTs. This analysis was individually performed for *TET*1, *TET*2, and *TET*3 in the same clinical samples examined in Figure 1C. Each expression level was normalized to that of *β-actin* in each sample. ***P < 0.0001; ns, non-significant. **E.** Quantitative real-time RT-PCR of *TET*1 in 80 GTs. **F.** Association between *TET*1 expression and regional lymph-node metastasis. GTs were divided into three groups according to whether cancer cells had spread to lymph nodes: N0, no regional lymph-node metastasis; N1, metastasis in one to two regional lymph nodes; N2, three to six; N3, seven or more. For panels C-F, the box plots show the median, 25th and 75th percentiles, and outliers. **G.** Kaplan-Meier analysis of patient survival based on *TET*1 expression in GTs. The log-rank test was used to compare survival between the low- and high-*TET*1 expression groups of patients based on average *TET*1 expression.

We found that the global level of 5hmC was consistently reduced in GTs compared with adjacent normal mucosa (NM), whereas global 5mC in the GTs was not lower by comparison (Figure 1A). NMs of all clinical samples immunostained positive for 5hmC, but staining was rarely detected in GTs including intestinal type and diffuse type (Figure 1B). Using ELISA, we further quantified global levels of 5mC and 5hmC in clinical samples. We established a standard curve for optical density (OD) at 450 nm for 5hmC and 5mC using known concentrations of 5hmC and 5mC (0.1-2 ng); *R*^2^ was calculated as 0.99 and 0.94, respectively (S1 Figure). Based on this *R*^2^, we estimated global levels of 5mC and 5hmC in 38 paired clinical tissues and found a significant decrease in 5hmC in GTs compared with corresponding NMs (*P* = 0.0033), whereas no significant difference was found in 5mC between GTs and NMs (*P* = 0.1776) (Figure 1C). This result suggested that global loss of 5hmC may be a general feature in primary GTs.

### *IDH*1/2 mutations are not found in GCs

We sequenced *IDH*1/2 in the GCs tested in this study to determine if previously identified *IDH*1/2 mutations are associated with decreased global 5hmC via modulation of TET1 activity. Sanger sequencing revealed homologous wild-type alleles for *IDH*1 (R132 site) and *IDH*2 (R172 site) in 10 GC cell lines (S2 Figure). Pyrosequencing also revealed no mutations in two hot spots in 38 GTs (S2 Table). Thus, the decrease in 5hmC may not correlate with *IDH*1/2 mutations, at least in the cancer specimens we tested.

### Correlation between global 5hmC level and *TET1* expression in primary GTs

Because the *TET* family converts 5mC to 5hmC [3], we examined which *TET* family gene correlated with the global decrease in 5hmC in primary GTs. Real-time quantitative reverse transcription (RT)-PCR of the same clinical samples showed that only *TET*1 mRNA level was significantly decreased in GTs compared with adjacent NMs (*P* < 0.001, left panel in Figure 1D); *TET*2 and *TET*3 were slightly but not significantly decreased in GTs, revealing that *TET*1 is more likely to be correlated with 5hmC biosynthesis in GCs. With a clinical sample size of 80 for real-time RT-PCR, we confirmed that *TET*1 mRNA level was indeed significantly decreased in GTs (Figure 1E, *P* < 0.0001). Table 1 lists clinicopathologic characteristics of patients regarding *TET*1 expression in GTs. According to the mean value of *TET*1 expression, we divided GT tissues into two groups, namely ‘*TET*1*_*high’ or ‘*TET*1*_*low’, and assessed potential correlations with clinicopathologic parameters. *TET*1*_*low was detected in 66% (53 of 80) of primary GTs. In particular, *TET*1_low was significantly more common in advanced GCs than in early GCs (*P* < 0.027) and in lymph node-positive compared with -negative metastases (*P* < 0.019), suggesting that *TET*1 loss of expression (LOE) may be a late event or an event that is associated with metastasis in the multistep process leading to gastric carcinogenesis.

**Table 1:** Status of *TET*1 expression level in gastric tumors with respect to clinicopathological characteristics

Characteristics		*TET*1 high (27/80)	*TET*1 low (53/80)	*P* value
Age (years)		58.07 ± 13.12	56.96 ± 13.87	0.7309^a^
Tumor size (cm)		5.39 ± 2.27	5.39 ± 2.05	0.9940^a^
Gender				
	Male	13	33	0.227^b^
	Female	14	20	
Histology				
	Intestinal	12	23	0.928^b^
	Diffuse	15	30	
Stage				
	EGC	5	2	0.027^c^
	AGC	22	51	
Lymph node metastasis				
	Negative	16	17	0.019^b^
	Positive	11	36	

All tumors were classified into two subtypes using average *TET*1 expression.

Tumor size was calculated based on the largest diameter measured for each tumor.

EGC, early gastric cancer; AGC, advanced gastric cancer.

a *P* value was measured with the Student's t test.

b Significance of association was determined using the χ^2^ test.

c Analyzed with Fisher's exact test.

Next, GTs were divided into subgroups based on whether there was concomitant lymph-node metastasis. No significant difference was found in *TET*1 expression between N0 (no proximal lymph-node metastasis), N1 (one to two proximal lymph-node metastases), N2 (three to six), and N3 (seven or more) GTs and NMs, but we detected a significant decrease in *TET*1 in N1-2 or N3 GTs compared with N0 GTs or NMs (Figure 1F). In addition, Kaplan-Meier survival analysis showed that *TET*1_low was significantly associated with poor survival of patients with GCs (Figure 1G, *P* = 0.0137).

### Global 5hmC level depends on *TET1* expression in GC cells

**Figure 2:**
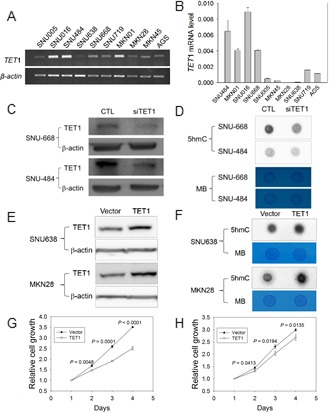
*TET1* siRNA analysis and *TET1* overexpression in GC cells **A.**, **B.** Analysis of *TET*1 mRNA level in 10 GC cell lines with RT-PCR. A. or quantitative real-time RT-PCR. B.*β-actin* was used as a control. **C.**, **D.** *TET*1 expression in knockdown cells using *TET*1 siRNA. **C.** Western blotting with SNU484 and SNU668 cells in which si*TET1* was transiently expressed. *β-actin* was used as a control (CTL). **D.** Dot blot assay with anti-5hmC using 700 ng DNA from the si*TET*1-treated cells in Figure 2C. **E.**, **F.** Experiment for *TET*1 overexpression.E. Western blotting after *TET*1 transfection into SNU638 and MKN28 cells. *β-actin* was used as a control. F.Dot blot assay with anti-5hmC and DNA from transfected cells in Figure 2E with 700 ng DNA. **G.**, **H.** Cell proliferation assay. Transfectants expressing vector only or the *TET*1 expression vector were cultured for 1-4 days, and then cell proliferation was measured using a CCK-8 kit (G, assay for SNU638; H, for MKN28). The data are cell index curves with mean ± SD from triplicate experiments. MB, methylene blue.

To examine whether the amount of 5hmC is associated with *TET*1 expression in GCs, we divided the GC cell lines into two groups based on *TET*1 expression as determined with RT-PCR (Figure 2A) or real-time RT-PCR (Figure 2B). *TET*1 mRNA level was high in 4 of 10 cell lines (SNU-016, SNU-484, SNU-668, MKN01) whereas the level was low in the remaining six lines (Figure 2B). We depleted *TET*1 in SNU-484 and SNU-668 cells via transfection with siRNA and investigated global TET1 level using western blotting. *TET*1-specific siRNA effectively knocked down TET1 in SNU-484 and SNU-668 cells (Figure 2C), and global 5hmC level was significantly decreased in these *TET*1-depleted SNU-484 and SNU-668 cells compared with controls, as determined by dot blotting (Figure 2D).

Next, we induced *TET*1 overexpression via transfection with the *TET*1 expression vector into SNU638 and MKN28 cells, in which *TET*1 is not expressed (Figure 2A). TET1 level was significantly elevated in both transfected cell lines compared with vector-transfected cells (Figure 2E), and global 5hmC was also increased in the transfected lines (Figure 2F). Thus, *TET*1 may play a crucial role in the biosynthesis of 5hmC in GC cells. Interestingly, the proliferation of *TET*1*-*induced cells was significantly decreased compared with vector-transfected cells at all culture times (Figure 2G), suggesting that *TET*1 downregulates the proliferation of GC cells.

### Methylation signature proximal to the *TET1* promoter region in a GT as assessed with genome-wide methylation profiling

**Figure 3:**
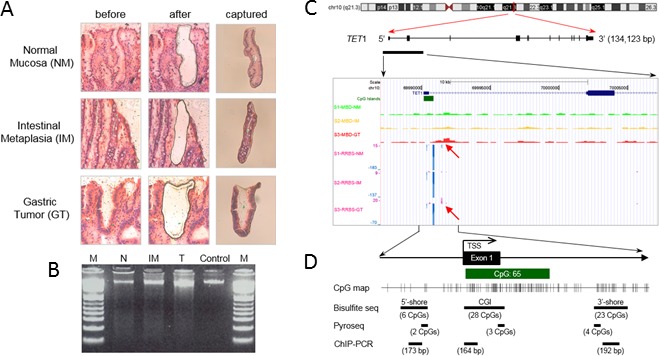
Methylation signatures proximal to the *TET1* promoter region detected with MBD-seq and RRBS **A.** LCM procedure. NM, IM, and GT cells were dissected from frozen tissue slides of one paired GT and its adjacent non-tumor tissue resected from a patient by gastrectomy. Left and middle columns indicate hematoxylin and eosin-stained tissues before and after LCM. The right column shows the captured tissues. **B.** Genomic DNA purified from LCM tissues was separated on a 1.5% agarose gel and detected with the GelRed method. High-molecular-weight DNA from blood was used as a control. M indicates DNA ladder. **C.** Methylation profiles near the *TET*1 promoter region in LCM DNAs. MBD-seq and RRBS were performed with DNAs from Figure 3B, and the result was visualized on a mirror site of the UCSC Genome Browser (hg ver.18). The upper three rows show peaks of the methylation enrichment score for NM (S1-MBD-N), intestinal metaplasia (S2-MBD-IM), and GT tissues (S3-MBD-T_adjusted) from MBD-seq. Lower rows show methylation signatures of NM (S1-RRBS-N), IM (S2-RRBS-IM), and GT tissues (S3-RRBS-T) from RRBS, showing methylated C (purple peaks, C read as C in CpG) or unmethylated C (blue peaks, T read as C in CpG) at single-nucleotide resolution. **D.** Schema of the experimental design for quantitative CpG methylation analysis and ChIP-PCR, which was performed for three regions: ‘CGI 5′-shore’, ‘CGI’, and ‘CGI 3′-shore’. Bisulfite sequencing assessed 6 CpGs from −639 to −226 in the CGI 5′-shore, 27 CpGs from −21 to +229 in the CGI, and 16 CpGs from +1026 to +1435 in the CGI 3′-shore. Pyrosequencing assessed 2 CpGs from −333 to −343 in the CGI 5′-shore, 3 CpGs from +178 to +199 in the CGI, and 4 CpGs from +1126 to +1148 in the CGI 3′-shore. ChIP-PCR was designed to amplify DNA fragments of 164 to 192 bp from each region.

To determine how *TET*1 mRNA level is regulated in GC cells, we first assessed the methylation status at CpG sites proximal to the *TET*1 promoter. We used laser capture microdissection (LCM) to prepare gastric normal mucosa (NM), intestinal metaplasia (IM), and GT cells that were prepared from frozen clinical tissue of one patient (Figure 3A). High-molecular-weight DNA was purified from each LCM tissue sample (Figure 3B), and Methyl-CpG Binding Domain Sequencing (MBD-seq) and Reduced Representation Bisulfite Sequencing (RRBS) were performed. After data processing and statistical testing, MBD-seq and RRBS revealed common methylation signatures in the downstream region of a CGI covering the *TET*1 promoter only in GC cells, whereas no signature was found in NM or IM cells (Figure 3C). The methylation signatures were detected at CpG sites within 0.8-2.2 kb downstream of the transcription start site (TSS) by MBD-seq or 1.3-1.4 kb downstream of the TSS by RRBS (Figure 3C). To determine whether these downstream signatures are common in GCs or whether a signature is present in another region such as the 5′-upstream region of the promoter or a CGI, we chose three regions for bisulfite sequencing or pyrosequencing: ‘5′-shore’ is the first region containing six CpG sites ranging from −639 to −226 bp from the TSS; ‘CGI’ is the second region of 28 CpG sites ranging of −21 to + 299 bp and overlapping with the CGI; ‘3′-shore’ is the third region of 23 CpG sites ranging from +1026 to +1435 bp (Figure 3D).

### Association between 3′-shore CpG methylation and *TET1* silencing in GC cell lines

**Figure 4:**
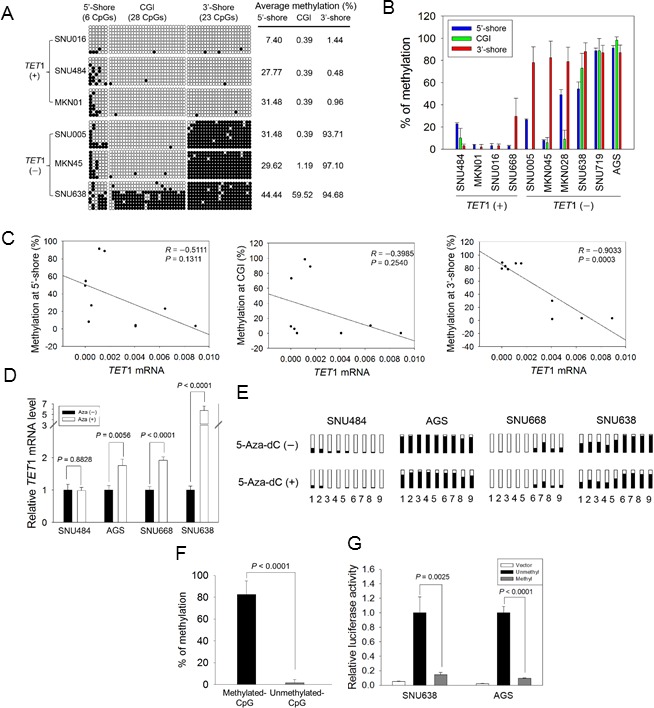
Association between CpG methylation proximal to the *TET1* promoter and its expression in GC cell lines **A.** Bisulfite sequencing of GC cell lines was performed for the three regions indicated in Figure 3D with three *TET*1-expressing (+) and another three *TET*1-silenced (−) cell lines. Open circles, unmethylated CpG sites; filled circles, methylated CpG sites. Each row represents a single clone. The numbers on the right represent the mean percentages of CpG sites that were methylated for each cell line. **B.** Pairwise comparison of CpG methylation with *TET*1 mRNA level in GC cell lines. CpG methylation in three regions and mRNA level were quantified for each cell line with pyrosequencing and real-time RT-PCR. Each experiment was performed in triplicate. **C.** Pearson's correlation analysis between mRNA level and CpG methylation in the 5′-shore (left panel), the CGI (middle), and the 3′-shore (right). This analysis was performed using the dataset in Figure 4B. **D.** Restoration of *TET*1 expression after 5-Aza-dC treatment. Real-time RT-PCR was used to examine four GC cell lines: SNU484, AGS, SNU668, and SNU638, before and after 5-Aza-dC treatment. Each value is the mean ± SD of three independent experiments. **E.** Comparison of CpG methylation status before and after 5-Aza-dC treatment. Pyrosequencing was performed for the CpG sites indicated in Figure 3D: 1 and 2, two CpGs from the 5′-shore; 3 to 5, three CpGs from the CGI; 6 to 9, four CpGs from the 3′-shore. Open squares indicate CpG sites that are fully unmethylated; black squares indicate various degrees of CpG methylation. **F.** Luciferase activity in the 3′-shore in GC cells. Methylated and unmethylated reporter constructs for 613 bp from the 3′-shore were established, and each methylation status was confirmed with pyrosequencing (left panel). Luciferase activity was examined in SNU638 and AGS cells, showing that the activity was significantly reduced in the methylated construct compared with the unmethylated construct (*P* = 0.002, *P* < 0.0001, respectively) (right panel). Each value is the mean ± SD of three independent experiments.

For bisulfite sequencing of GC cell lines, we selected three *TET*1 high-expression cell lines (SNU016, MKN01, SNU484; ‘*TET*1 (+)’) and three *TET*1 low-expression cell lines (SNU005, MKN45, SNU638; ‘*TET*1 (−)’) based on quantitative real-time RT-PCR (Figure 2B). Within the 5′-shore, bisulfite sequencing showed that the mean methylation frequency in *TET*1 (+) cells (22.22%) was lower than that in *TET*1 (−) cells (35.18%), but the difference was not statistically significant (Figure 4A). In the CGI region, five GC cell lines showed a methylation-free status, ranging from 0.39 to 1.19, except for SNU638 (59.5%); the difference was also not statistically significant. On the other hand, the mean methylation frequency in *TET*1 (+) cells (0.96%) in the 3′-shore region was quite different from that in *TET*1 (−) cells (95.16%), indicating that the CpG sites in the 3′-shore are critical methylation sequences associated with *TET*1 silencing (Figure 4A). Further pyrosequencing of these three regions (Figure 4B) also showed that loss of *TET*1 expression was associated with CpG methylation in the 3′-shore (*r* = −0.9033, *P* = 0.0003; right panel in Figure 4C).

To confirm that the 3′-shore CpG methylation was associated with *TET*1 silencing, GC cell lines having various methylation levels in the 3′-shore were treated with 5-aza-2′- deoxycytidine (5-Aza-dC). Pretreatment values were as follows: SNU484, methylation-free (2.25 ± 0.95%); SNU668, weakly methylated (29.66 ± 7.71%); AGS and SNU638, heavily methylated (87.01 ± 6.73% and 92.26 ± 2.41%). Quantitative real-time RT-PCR showed that *TET*1 expression was significantly increased or restored in AGS (*P* = 0.0056), SNU668 (*P* < 0.0001), and SNU638 (*P* < 0.0001) cells after 5-Aza-dC treatment, whereas no difference was observed before and after 5-Aza-dC treatment in SNU-484 cells (*P* = 0.8828, Figure 4D). Methylation analysis revealed that methylation before 5-Aza-dC treatment was slightly lower at all CpG sites of the cell lines tested compared with after drug treatment (Figure 4E). SNU484, which lacks methylation on the 3′-shore, showed no change in *TET*1 expression after drug treatment, whereas SNU668, which is weakly methylated in the 3′-shore, showed decreased CpG methylation in the same region and restoration of *TET*1 expression. These data demonstrated that *TET*1 expression is relevant to CpG demethylation on the *TET*1 3′-shore*.*

To assess the effect of CpG methylation in the 3′-shore, we performed a luciferase assay with SNU638 or AGS cell lines transfected with CpG-methylated or CpG-unmethylated reporter constructs. Figure 4F shows the methylation status for the construct methylated by *Sss*I methylase and S-adenosylmethionine and for the unmethylated control construct. Luciferase activity was greatly decreased in the methylated reporter construct compared with the unmethylated reporter in both transformed SNU638 and AGS cells (Figure 4G) (*P* = 0.0025 and *P* < 0.0001, respectively), strongly indicating that CpG methylation in the 3′-shore is critical for controlling *TET*1 expression in the GC cell lines we examined.

### Association between *TET1* 3′-shore CpG methylation and decreased *TET1* expression in primary GTs

**Figure 5:**
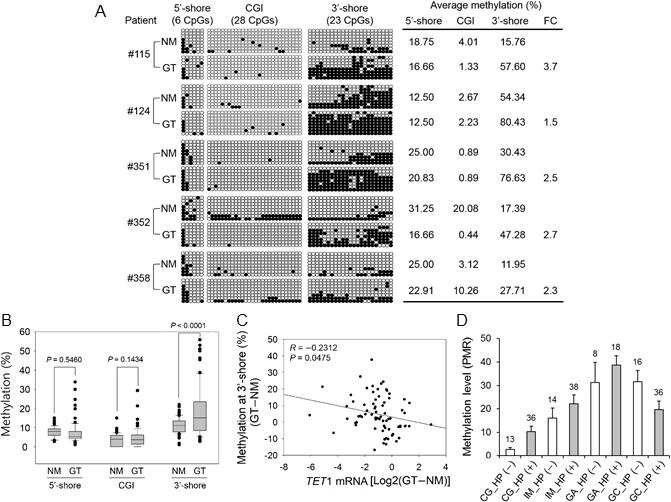
Association between CpG methylation in the CGI 5′-shore, CGI, and CGI 3′-shore and *TET1* expression in primary GTs **A.** Bisulfite sequencing was performed for three regions in five paired primary GTs and adjacent NMs. The description for this figure is the same as that of Figure 4A. Asterisks on the right indicate a significant increase in CpG methylation in the 3′-shore in primary GTs compared with the adjacent NMs. FC, fold change. **B.** Pyrosequencing of three regions with 80 paired primary GTs and NMs. The box plots show the median, 25th and 75th percentiles, and outliers. **C.** Pearson's correlation analysis between the 3′-shore CpG methylation and *TET*1 mRNA level. This analysis was performed with 80 clinical samples with both expression (Figure 1E) and methylation (Figure 5B) data. The *y* axis indicates the methylation difference between paired normal and tumor tissues (that is, GT − NM). Relative expression values are the log2 ratio of GT to NM. **D.** Gradual methylation in the CGI 3′-shore during gastric carcinogenesis. MethyLight analysis was performed with 179 FFPE tissue samples of multistep gastric lesions including *H. pylori*-negative [HP (−)] and *H. pylori*-positive [HP (+)] tissues. Numbers above each vertical bar indicate the number of tissues tested in this analysis. Open bar, HP (−); gray bar, HP (+). The mean methylation level within each group is shown as a percent of methylated reference on the *y* axis.

To evaluate the clinical significance of *TET*1 in GC, we also examined methylation status in 80 paired GTs and their NMs. Bisulfite sequencing revealed that the mean methylation in the 5′-shore or CGI did not differ greatly between tumor and NMs from five patients (Figure 5A). However, CpG methylation in the 3′-shore was significantly greater in GTs (57.93 ± 21.70%, n = 5) compared with NMs (25.97 ± 17.31%, n = 5) (*P* < 0.05). This result corresponded well with the pyrosequencing results from 80 clinical tissues. That is, CpG methylation in the 3′-shore was significantly increased in GTs compared with NMs (*P* < 0.0001), whereas no significant difference was detected between GTs and NMs in the 5′-shore or CGIs (Figure 5B). CpG methylation in the 3′-shore correlated negatively with *TET*1 mRNA level (*r* = −0.2312, *P* = 0.0475, Figure 5C).

### CpG methylation at the *TET1* 3′-shore during gastric carcinogenesis

To determine the methylation status at the *TET*1 3′-shore during gastric carcinogenesis, we performed MethyLight analysis of 179 formalin-fixed paraffin-embedded (FFPE) samples of various types of gastric lesions, including chronic gastritis (CG, without IM), IM, gastric adenoma (GA), and GC. *TET*1 methylation (>4% compared with the methylated reference) was found in CG, IM, GA, and GC at frequencies of 57.1%, 82.7%, 100%, and 75%, respectively (Chi-square test, *P* < 0.001). *TET*1 methylation differed significantly in CG depending on *Helicobacter pylori* infection, but no significant difference was found in *TET*1 methylation level in IM, GA, or GC with and without *H. pylori* infection (Figure 5D). This result suggested that *TET*1 3′-shore CpG methylation is initiated at an early stage such as IM or CG with *H. pylori* infection during gastric carcinogenesis and tends to accumulate as carcinogenesis progresses.

### Association between bivalent chromatin structure and transcriptional repression at the *TET1* 3′-shore region

**Figure 6:**
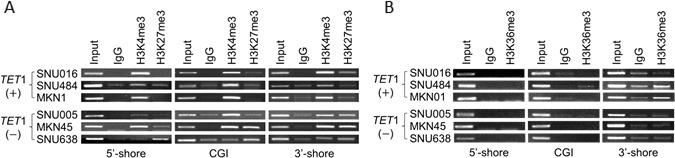
Histone modifications at *TET1* genomic loci in GC cell lines **A.**, **B.** ChIP-PCR to detect histone modifications in the CGI 5′-shore, CGI, and 3′-shore regions of *TET*1. Immunoprecipitated DNAs and input DNAs were derived from three *TET*1-expressing (+) and another three *TET*1-silenced (−) cell lines, as shown Figure 4A, using anti-H3K4me3 and anti-H3K27me3 A.or H3K36me3B. IgG ChIP DNA samples were amplified using the same primers as the negative control. The amplified DNA was run on a 1.2% agarose gel. The data are representative of three independent experiments of triplicate samples.

To determine whether the presence of histone modifications near the *TET*1 promoter region was associated *TET*1 mRNA level or CpG methylation, we used chromatin immunoprecipitation (ChIP)-coupled PCR to assess transcriptionally activating (H3K4me3) and repressive (H3K27me3) histone marks in the 5′-shore, CGI, and 3′-shore regions (Figure 3D). We detected H3K4me3 in all three regions of *TET*1 (+) cells (SNU016, SNU484, and MKN01) in which *TET*1 mRNA was detected (Figure 2B), whereas H3K27me3 was not detected in those lines (Figure 6A, upper panel). On the other hand, *TET*1 (−) cells (SNU005, MKN45, and SNU638) in which *TET*1 transcription was repressed (Figure 2B) showed various combinations of active and repressive marks per region or cell line. H3K4me3 was detected in the CGIs of SNU005 cells, whereas the bivalent mark [19] of H3K4me3 and H3K27me3 was observed in the 3′-shore. In MKN45 cells, the bivalent mark was detected in both the CGI and 3′-shore, but only H3K4me3 was detected in the 5′-shore. In SNU638, the bivalent mark was detected in the 3′-shore, whereas H3K27me3 was observed in both the 5′-shore and CGI. Thus, this bivalent mark was strongly associated with extensive CpG methylation in the 3′-shore and was a common feature among cell lines in which *TET*1 transcription was repressed, suggesting that epigenetic alteration, including DNA methylation and histone modification in the 3′-shore region, may play a detrimental role in repressing *TET*1 transcription regardless of the open chromatin structure in the CGI of SNU005 cells or in the 5′-shore of MKN45 cells. Because the 3′-shore is in the gene body region, we also performed ChIP to assess whether the H3K36me3 mark could predict *TET*1 transcriptional activity. Figure 6B shows that H3K36me3 was observed only in the 3′-shore of SNU484 and MKN01 cells of the three *TET*1 (+) cell lines in which *TET*1 was transcribed and was never detected in the 3′-shore of any *TET*1 (−) cell line.

## DISCUSSION

The recent finding that oxidation of 5mC to 5hmC by *TET* family proteins occurs in mammalian genomes has suggested that DNA methylation may be reversible in mammalian cells [3]. However, the role of this type of DNA modification in epigenetic regulation during human carcinogenesis and the mechanistic details of this process have not been clearly demonstrated. In this study, all techniques including dot-blot, immunohistochemistry, and ELISA consistently showed that 5hmC levels were reduced in GTs compared with adjacent NMs, similar to previous reports on various solid tumors including GTs [6, 12, 20-22]. To elucidate the possible mechanism underlying the decrease in 5hmC in GTs, we first examined *IDH*1/2 mutations, which are associated with decreased 5hmC via modification of *TET* activity [23]. However, we found no mutations at hot spots such as the R132 site of *IDH*1 or the R172 site of *IDH*2 in 10 GC cell lines and 38 GTs tested, indicating that mutations at these known hot spots of *IDH*1/2 are very rare—at least in the GC cells and GTs we tested. On the one hand, a recent study showed that mutations in fumarate hydratase (*FH*) and succinate dehydrogenase (*SDH*) detected in various cancers are associated with 5hmC level [24]. Therefore, further studies must address whether *FH* and *SDH* are mutated in GCs and the mutations are associated with decreased 5hmC level in GCs.

We observed that *TET*1 was the only *TET* gene for which expression was significantly decreased in GTs compared with matched NMs, as previously reported [12]. In contrast to our present results, a recent study showed that all three *TET* genes were significantly downregulated in patients with GC [25]. Because the sample size of that study did not differ significantly from that of our present study, this discrepancy may be due to the use of different normalizing genes rather than sampling error. That is, we used *β-actin* for normalization whereas Du et al. [25] used *GAPDH*. Notably, expression of *GAPDH* is more affected than that of *β-actin* during tumorigenesis [26-28]. Further analysis with a cohort of GTs revealed that *TET*1 LOE was significantly more common in advanced GC than in early GC and in metastasis-positive lymph nodes compared with metastasis-negative lymph nodes, suggesting that *TET*1 LOE may be a late event or an event that is associated with metastasis in the multistep process leading to gastric carcinogenesis. Importantly, we found a significant association between *TET*1 LOE and poor survival in patients with GC, increasing the diagnostic and prognostic value for patients with GC and suggesting substantial clinical relevance.

siRNA-mediated knockdown of *TET*1 in SNU-484 and SNU-668 cells resulted in a significant decrease in global 5hmC level. Also, *TET*1 overexpression in AGS cells resulted in a significant increase in 5hmC level, and the cells grew more slowly than control cells, indicating that *TET*1 may modulate DNA methylation and have anti-proliferative activity in GC cells. These results are consistent with the previous finding that *TET*1 acts as a tumor suppressor gene to regulate critical pathways involved in cell proliferation and metastasis [29, 30].

Although a decrease in 5hmC level in *TET*1 is associated with its LOE in various cancers including GC, the mechanism by which *TET*1 is suppressed in solid tumors has not been elucidated. A recent study showed that *TET*1 expression is negatively regulated by CpG methylation near the TSS in breast cancer cells and T-cell acute lymphoblastic leukemia cell lines [31]. However, we found no difference in *TET*1 CGI methylation between GTs and matched NMs (data not shown). It is interesting that our MBD-seq and RRBS analysis revealed prominent methylation signatures in tumor cells only in the 3′-shore and not in CGIs or the 5′-shore, suggesting that *TET*1 transcription may be regulated by methylation in the 3′-shore but not CGIs or the 5′-shore in GCs. Although DNA methylation of CGIs in promoters results in transcriptional silencing [32], only ∼70% of human gene promoters contain CGIs [33], and only 6.8% of CpGs in the human genome reside within CGIs [34]. Therefore, many potentially informative CpG sites remain to be examined. A recent finding showed that DNA methylation can directly silence genes without CGIs in their promoters or CGI shores [35]. A more recent study showed that DNA methylation within 3 kb downstream of the TSS is consistently linked with gene downregulation regardless of hyper- or hypomethylation upstream of the TSS [36]. Based on this finding, we suggest that *TET*1 in GCs may be one gene in a cluster in which the 3′-shore is methylated in a cancer-specific pattern.

In this study, bisulfite sequencing and pyrosequencing revealed that *TET*1 downregulation was significantly associated with heavy methylation in the 3′-shore but not the 5′-shore or the CGI in six *TET*1 (¯) GC cells (SNU005, MKN45, MKN28, SNU638, SNU719, and AGS). Moreover, *TET*1 expression was restored in SNU638 and AGS cells, and even in SNU668 cells in which only the CGI 3′-shore was slightly methylated after 5-Aza-dC treatment. Interestingly, no change in *TET*1 expression before drug treatment compared with after treatment was seen in SNU484 cells in which the CGI 3′-shore was unmethylated. Furthermore, a luciferase assay of methylated and unmethylated constructs of a 613-bp fragment of the 3′-shore showed that activity was greatly decreased in the methylated construct, suggesting that methylation in the 3′-shore plays a critical role in repressing *TET*1 expression in GC cells. This result was confirmed by an analysis of clinical tissues, which revealed that methylation was significantly increased only in the 3′-shore in 83 GTs compared with NMs, showing a negative correlation between CGI 3′-shore methylation and *TET*1 expression. Thus, our data indicate that the 3′-shore of *TET*1 is a critical region for cancer-specific methylation for transcriptional regulation in primary GTs as well as GC cell lines. However, we cannot rule out the possibility that other unidentified genes or factors that are epigenetically silenced in GC cells but are activated by 5-Aza-dC are involved in regulating *TET*1. This aspect will require further study.

Previous findings have shown that promoter methylation in many genes occurs in non-cancerous tissues adjacent to GTs [37, 38] and even in non-neoplastic gastric mucosa associated with *H. pylori*, regardless of GTs [39, 40]. Thus, overall deregulation of the DNA methylation machinery may be an early event in gastric carcinogenesis and may become more severe as the cancer progresses. In this study, additional MethyLight analysis with 179 FFPE samples showed that CpG methylation in the 3′-shore of *TET*1 was initiated at an early stage such as IM or CG + *H. pylori* infection and that methylation accumulated during carcinogenesis, indicating that *H. pylori* predisposes tissue to carcinogenesis, as shown previously [41].

Transcriptional silencing is dependent on chromatin structure, which is regulated by cross-talk between DNA methylation patterns and histone modifications. If the 3′-shore is critical for regulating transcription as described in this study, the methylation signature in the 3′-shore should be compatible with histone marks. Our study revealed that the bivalent mark H3K4me3+H3K27me3 was strongly associated with heavy CpG methylation in the 3′-shore of *TET*1 in GC cell lines, in which *TET*1 transcription was repressed, supporting previous reports that such bivalent marks are common in adult tumor cells and lead to transcriptional silencing [17, 18]. The presence of dense DNA methylation approximately 1 kb downstream of the TSS may be associated with a modest decrease in elongation efficiency [42]. Based on this model, we therefore suggest that *TET*1 transcription is mainly inhibited at the elongation step via cross-talk between dense DNA methylation and bivalent histone marks at the CGI 3′-shore.

Our results demonstrate that 5hmC is decreased in GCs and is directly associated with *TET*1 LOE, which is associated with dense methylation coupled with bivalent histone marks in the 3′-shore. Therefore, our study indicates that epigenetic features in the 3′-shore of *TET*1 may decrease the efficiency of transcription by inducing formation of a compact chromatin structure. Finally, DNA methylation in the 3′-shore of *TET*1 may be one mechanism underlying 5hmC loss in GC.

## MATERIALS AND METHODS

### Cell lines and tissue samples

The 10 GC cell lines SNU005, −016, −484, −638, −668, −719, MKN01, −28, −45, and AGS used in this study were purchased from the Korean Cell Line Bank (http://cellbank.snu.ac.kr/index.htm). All cell lines were maintained in RPMI-1640 medium (WelGENE Inc., Seoul, Korea) supplemented with 10% FBS and 1% antibiotic-antimycotic solution (Invitrogen, Carlsbad, CA) in a humidified atmosphere containing 5% CO_2_ at 37°C. Eighty paired human GTs and adjacent NMs were obtained from the Hanyang University Hospital, Seoul, Korea. Patients comprised 46 men and 34 women with a mean age of 57.33 ± 13.54 years. The study protocol was approved by the Institutional Review Board of Hanyang University Hospital, and all data and specimens were collected after obtaining written informed consent from patients.

### LCM

To obtain highly homogeneous cells for methylome analysis, paired specimens of GT tissue and adjacent non-tumor tissue were harvested immediately after surgical resection from a patient with GC and embedded in TissuTek OCT medium (Sakura, Tokyo, Japan) followed by storage at −80°C. The frozen tissues were cut at 8 μm thick, mounted onto membrane slides for photoactivated localization microscopy (PALM) (Zeiss, Munich, Germany), and stained with hematoxylin and eosin. Each specimen was covered with a liquid to improve the optical resolution of tissue sections. LCM was performed using a PALM system (Zeiss). The NM, IM, and GT cells were identified using PALM Robosoftware, and for this purpose cells were placed into 0.5-ml Adhesive-Cap tubes using the PALM system. DNA was extracted using the QIAamp DNA Micro kit (Qiagen, Valencia, CA). After purification, genomic DNA was separated on a 0.8% agarose gel and stained with GelRed (Biotium). The concentration of genomic DNA was quantified using the PicoGreen dsDNA Quantitation kit (Molecular Probes, Eugene, OR).

### Dot blot assay

To determine the global levels of 5mC and 5hmC in clinical tissues, 2 μl of DNA (200 or 400 ng/μl) from each sample was denatured and spotted onto a positively charged nylon-based membrane (Amersham Biosciences, Freiburg, Germany), which was cross-linked and blocked with 5% non-fat milk. After washing in 0.1% Tween 20 in PBS, the membrane was incubated with anti-5hmC (1:10,000, Active Motif, Carlsbad, CA) and anti-5mC (1:1000, Abcam, Cambridge, UK) overnight at 4°C followed by incubation with horseradish peroxidase-conjugated anti-rabbit IgG secondary antibody (Santa Cruz Biotechnology, 1:5000). The signal was developed with WEST-ZOL Plus (Intron) and visualized using RAS-4000 (Fujifilm, Tokyo, Japan). Relative 5hmC intensity was calculated by dividing the positively stained areas by the total area using Multi Gauge v3.0 (Fujifilm). To estimate the relative concentration of DNA, we performed methylene blue staining (0.02% methylene blue in 0.3 M sodium acetate, pH 5.2).

### Immunohistochemistry

To examine the presence and distribution of the 5hmC signature in clinical tissues, FFPE sections were dewaxed and rehydrated through a graded series of ethanol followed by blocking of endogenous peroxidase activity for 15 min. Slides were then washed in PBS, and antigen retrieval was performed in a citrate buffer solution with microwaving for 10 min. Slides were then incubated with anti-5hmC (1:10,000; Active Motif) or anti-5mC (1:1000; Abcam) for 1 h at room temperature. Sections were then incubated with biotinylated secondary antibody and detected using the ChemMate Envision detection kit (Dako, Carpinteria, CA).

### ELISA

To determine global levels of 5mC and 5hmC in clinical samples, we performed ELISA using a MethylFlash Methylated and Hydroxymethylated DNA Quantification kit (Epigentek, NY, USA). The reference DNA fragments containing 5mC, 5hmC, and cytosine were used as positive and negative standards. A standard curve was constructed using various 5mC and 5hmC concentrations. The terminal OD was read on a Benchmark Plus microplate reader (Bio-Rad, Hercules, CA). The amount of 5hmC and 5mC is proportional to OD. After subtracting negative control readings from the sample and the standard readings, the value for 5hmC or 5mC in each sample was calculated as a ratio of sample OD to standard OD.

### Mutation analysis of *IDH*1/2

To examine the presence of *IDH*1^R132^ or *IDH*2^R172^, which are known mutation hotspots [10], we performed Sanger sequencing with GC cell lines or pyrosequencing with the single-nucleotide polymorphism mode for the GT tissues. S1 Table lists all primer sequences and conditions for Sanger sequencing and pyrosequencing.

### Quantitative real-time RT-PCR

To quantify the expression of *TET* family members in GC lines and clinical tissue samples, 2 μg total RNA from each sample was reverse transcribed into cDNA using iScript reverse transcriptase (Bio-Rad). Real-time RT-PCR was performed using iQ SYBR Green Supermix (Bio-Rad) with a CFX96 real-time PCR system (Bio-Rad). mRNA levels were normalized to levels of *β-actin*. Table S1 describes all primer sequences and conditions.

### Transfection with siRNA

Double-stranded siRNA oligonucleotides targeting *TET*1 were purchased from Bioneer (catalog no. 1038120; Daejeon, Korea). Each siRNA oligonucleotide (1 μM, in solution T from Bioneer) was transfected into SNU-484 or SNU-668 cells using Nucleofector (Amaxa Biosystems, Cologne, Germany). Knockdown of TET1 was confirmed with western blotting using anti-TET1 (1:250, GeneTex, Irvine, CA). β-actin (1:500; Sigma, St. Louis, MO, USA) served as a control in the same samples. Horseradish peroxidase-conjugated anti-mouse IgG (1:5000, Santa Cruz Biotechnology) was used as the secondary antibody.

### Establishment of the *TET*1 expression vector and transfection of cancer cells

Human *TET*1 cDNA was amplified from the *TET*1 clone (Fisher Scientific, Pittsburgh, PA), and the PCR product was subcloned into the *Nhe*I/*Xba*I sites of pcDNA 3.1(+) (Invitrogen). The *TET*1 sequence was verified with Sanger sequencing. For *TET*1 transfection of AGS cells, we used Lipofectamine Plus (Invitrogen). TET1 and 5hmC levels were confirmed with western blotting and dot blots, respectively. Western blotting was performed as described [43]. For the cell proliferation assay, we used the Cell-Counting Kit-8 (Dojindo, Kumamoto, Japan). At each time point (28-96 h), we added CCK-8 solution to the wells, incubated the solution at 37°C for 2 h, and measured OD (450 nm) using a spectrophotometer (Bio-Rad).

### MBD-seq library construction and sequencing

LCM was used to isolate NM, IM, and GT cells, and cellular DNA was purified and then fragmented to 100-500 bp with gas at 44 psi for 1 min through a nebulizer (Agilent Technologies, Santa Clara, CA) and then enriched for methylated DNA using the MethylMiner Methylated DNA Enrichment kit (Invitrogen). Briefly, methylated DNAs were precipitated from 500 ng fragmented DNAs via binding to the methyl-CpG binding domain of human MBD2, which was coupled to magnetic Dynabeads. The methylated fragments were then eluted with High-Salt Elution Buffer (Invitrogen) and purified using the MinElute PCR Purification kit (Qiagen). The methylated DNA fragments were ligated to one pair of adaptors (S1 Table) for sequencing, size-fractioned on a 2% agarose gel to yield 200- to 300-bp fragments, and subjected to 18 cycles of PCR using primers described in Table S1. Each library was diluted to 8 pM for 76 cycles of single-read sequencing on the Illumina Genome Analyzer II.

### RRBS library construction and sequencing

RRBS was performed as described [44-46]. Briefly, LCM DNAs (300 ng each) were digested with *Msp*I (NEB, Ipswich, UK), cleavage by which is independent of CpG methylation status. Digested DNAs were ligated to adaptors for RRBS (S1 Table) that were synthesized using methyl cytosine to prevent sequence changes during bisulfite modification in the next step and size-fractionated to obtain DNA fragments of 40-120 bp and 120-220 bp. After two successive rounds of bisulfite treatment, 18 cycles of PCR were performed to construct the bisulfite-converted library. Then each library was diluted to 8 pM, and 76 cycles of single-read sequencing were performed with the Illumina Genome Analyzer II.

### Base-calling procedure

Base calling with MBD-seq data was performed throughout the routine procedure of the Illumina pipeline module, bclConverter v1.7, with 76 single-read cycles. The sequences were aligned with human genome assembly, hg18, using ELAND version 2 with default parameters. To evaluate the methylation peak signature, the aligned, coordinated sequences were extended up to 200 bp from the start position. Next, the coverage depth of methylated reads was calculated at 200-bp resolution. These calculated count values were converted into methylation enrichment scores to remove the bias among the amount of reads from different samples. Adjusted methylation enrichment scores were exported as a BED file and visualized with our mirror of the UCSC Genome Browser owing to the large size. To identify differentially methylated regions in the MBD-seq data, the sliding-window approach was applied to find methylation differences of >2-fold between samples (NM vs. IM or NM vs. GT) within a 1-kb range with a 200-bp bin shift (*t*-test, *P* < 0.01). For RRBS, the base-calling procedure was the same as with MBD-seq, but a mapping step was performed with the methylation-specific mapping tool BRAT [47] for short bisulfite-treated reads because unmethylated cytosines within sequences read from RRBS are converted to thymines. Methylated and unmethylated CpGs were counted, and an enrichment test (Fisher's exact test) was used to calculate methylation frequency during gastric carcinogenesis with a 2 × 3 contingency table. After the frequency test, Bonferroni correction criteria regarding all CpG sites sequenced with RRBS were applied to reduce type 1 errors. Then, the differentially methylated regions with three or more significant CpG sites were selected as prospective candidates.

### Bisulfite sequencing

Six CpG sites in the CGI 5′-shore, 28 CpG sites in CGI, and 23 CpG sites in the CGI 3′-shore of *TET*1 were selected for bisulfite sequencing (Figure 3D). Genomic DNA (1 μg) was modified using the EZ DNA Methylation-Gold kit (Zymo Research, Orange, CA). Bisulfite-modified DNA was amplified using a specific primer set targeted to the region of interest, and PCR products were cloned into pGEM-T Easy (Promega, Southampton, UK). S1 Table lists primer sequences for bisulfite sequencing.

### Pyrosequencing

Two CpG sites in the CGI 5′-shore, three CpG sites in CGIs, and four CpG sites in the CGI 3′-shore of *TET*1 were selected for quantitation of methylation (Figure 3D). Bisulfite-modified DNA (100 ng) was amplified with PCR in a 25-μl reaction using 2× Dye Mix polymerase (Enzynomics, Daejeon, Korea) to yield 153 bp for the CGI 5′-shore, 318 bp for CGIs, or 373 bp for the CGI 3′-shore using the primer sets shown in S1 Table. PCR was performed using an initial melting step of 95°C for 1 min followed by 50 cycles of 95°C for 30 s, 56°C for 40 s, and 72°C for 40 s, with final incubation at 72°C for 5 min. Pyrosequencing was performed as described [48, 49] with a sequencing primer for each region on the PSQ HS 96A System (Biotage AB, Kungsgatan, Sweden). S1 Table lists primer sequences and conditions used for pyrosequencing.

### MethyLight analysis

Using the MethyLight assay [50], the methylation status at the *TET*1 CGI 3′-shore was examined in 179 FFPE tissue samples of various types of gastric lesions, including chronic gastritis (without IM), intestinal metaplasia, GA, and GT. Glass slides containing these samples were viewed under a light microscope, and lesions were marked and dissected manually. S1 Table lists nucleotide sequences of the oligonucleotide primers and probes.

### Treatment of cells with 5-Aza-dC

GC cells were seeded in 10-cm dishes at a density of 1 × 10^6^ cells at 1 day before drug treatment and were then treated with 2 μM 5-Aza-dC (Sigma-Aldrich, Taufkirchen, Germany) every 48 h for 4 days.

### Luciferase reporter assay

To assess any functional role for CpG sequences of interest, the target sequence (613 bp) was amplified from 69,991,078 to 69,991,690 of human chromosome 10 (hg18) using the primer set described in Table S1 and inserted into the *Avr*II/*Spe*I sites of the pCpGfree-promoter-Lucia vector (InvivoGen, Toulouse, France), which is a reporter plasmid completely devoid of CpG dinucleotides. For *in vitro* methylation, 10 μg of the reporter constructs was incubated with 32 U *Sss*I methylase (NEB) and 32 mM S-adenosylmethionine (NEB) at 37°C overnight. After confirmation of the methylation status of methylated or unmethylated constructs with pyrosequencing, luciferase activity was measured using QUANTI-Luc (InvivoGen) and normalized to that of *Renilla* luciferase (Promega). The experiment was performed in triplicate.

### ChIP

ChIP was performed using a ChIP assay kit (Upstate Biotechnology, Lake Placid, NY) according to the manufacturer's protocol with some modifications. After cross-linking of protein to DNA and shearing of DNA strands with sonication, each supernatant was pre-cleared with pre-chilled *Staphylococcus aureus* cells, diluted in ChIP dilution buffer, and immunoprecipitated with 2 μg normal rabbit IgG (Millipore, catalog no. 12-370), 2 μg anti-H3K4me3 (Millipore, catalog no. 04-745), 5 μg anti-H3K27me3 (Millipore, catalog no. 07-449), 2 μg anti-H3K36me3 (Abcam, catalog no. ab9050), or no antibody. Immunoprecipitated DNA was recovered using the QIAquick PCR Purification kit (Qiagen) and used to amplify 173 bp from the CGI 5′-shore, 192 bp from the CGI 3′-shore, and 164 bp from CGIs with PCR in a 15-μl volume containing SYBR Premix EX Taq (Takara, Tokyo, Japan) and specific primer sets (S1 Table). Fragments were analyzed with agarose gel electrophoresis.

### Statistical analysis

For the analysis of global levels of 5mC or 5hmC, mRNAs, and DNA methylation from the 80 patients, we used the Student's *t*-test to evaluate the significance of differences between GTs and adjacent NMs. To examine the effect of the *TET1* expression (in the high- and low-mRNA expression groups) on patient survival, we performed Kaplan-Meier survival analysis and the log-rank test using R software (version 2.6.1). The correlation between *TET1* mRNA level and DNA methylation was determined using Pearson's correlation coefficient (*R*). Results for which *P* was <0.05 were considered statistically significant.

### Data access

The MBD-seq data from this study were submitted to the NCBI Gene Expression Omnibus (GEO; http://www.ncbi.nlm.nih.gov/geo/) under the reference series GSE55160 with subseries of GSM1330609_1154337-MBD-GM for S1-MBD-N, GSM1330610_1154337-MBD-IM for S2-MBD-IM, and GSM1330611_1154337-MBD-adjustedGC for S3-MBD-T_adjusted, and the RRBS data under the reference series GSE55159 with subseries of GSM1330612_1154337-RRBS-GM for S1-RRBS-N, GSM1330613_1154337-RRBS-IM for S2-RRBS-IM, and GSM1330614_1154337-RRBS-GC for S3-RRBS-T.

## SUPPLEMENTARY MATERIAL FIGURES AND TABLES


