# Supplementary material for: Decrease of 5hmC in gastric cancers is associated with TET1 silencing due to with DNA methylation and bivalent histone marks at TET1 CpG island 3′-shore
Source: Oncotarget. 2015 Oct 10;6(35):37647–62. doi: 10.18632/oncotarget.6069 (PMC4741955; doi:10.18632/oncotarget.6069)
Supplement: Supplementary file 1 [file oncotarget-06-37647-s001.pdf]

Decrease of 5hmC in gastric cancers is associated with *TET1* silencing due to with DNA methylation and bivalent histone marks at *TET1* CpG island 3′-shore

Supplementary Material

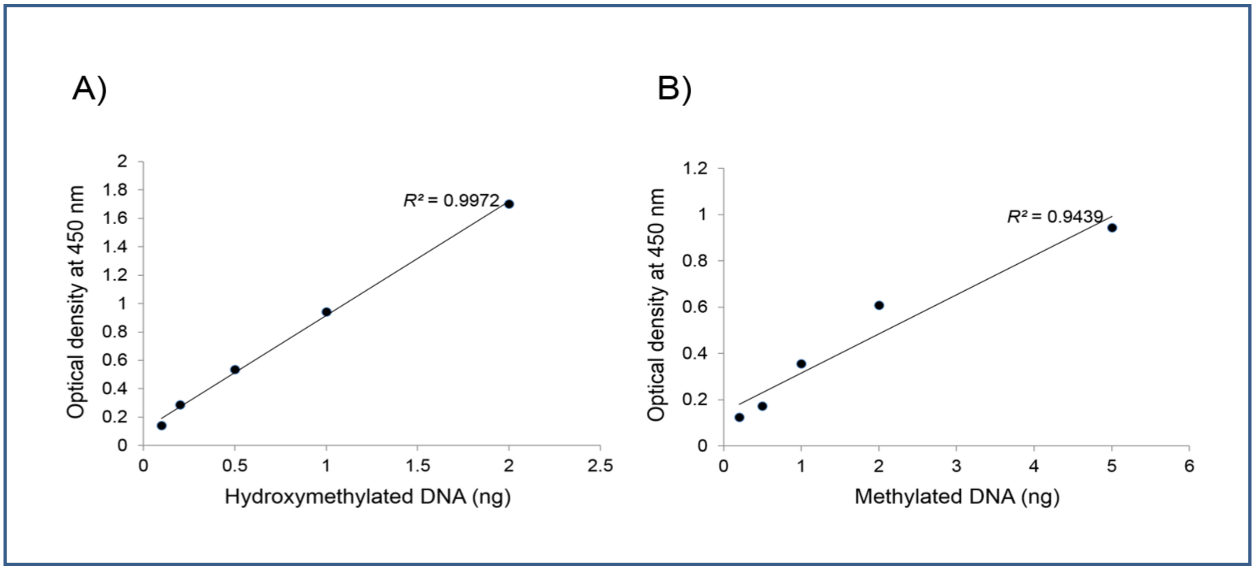

S1 Figure. Standard curve for ELISA to quantify 5hmC (A) and 5mC (B) levels in paired primary GT tissues.

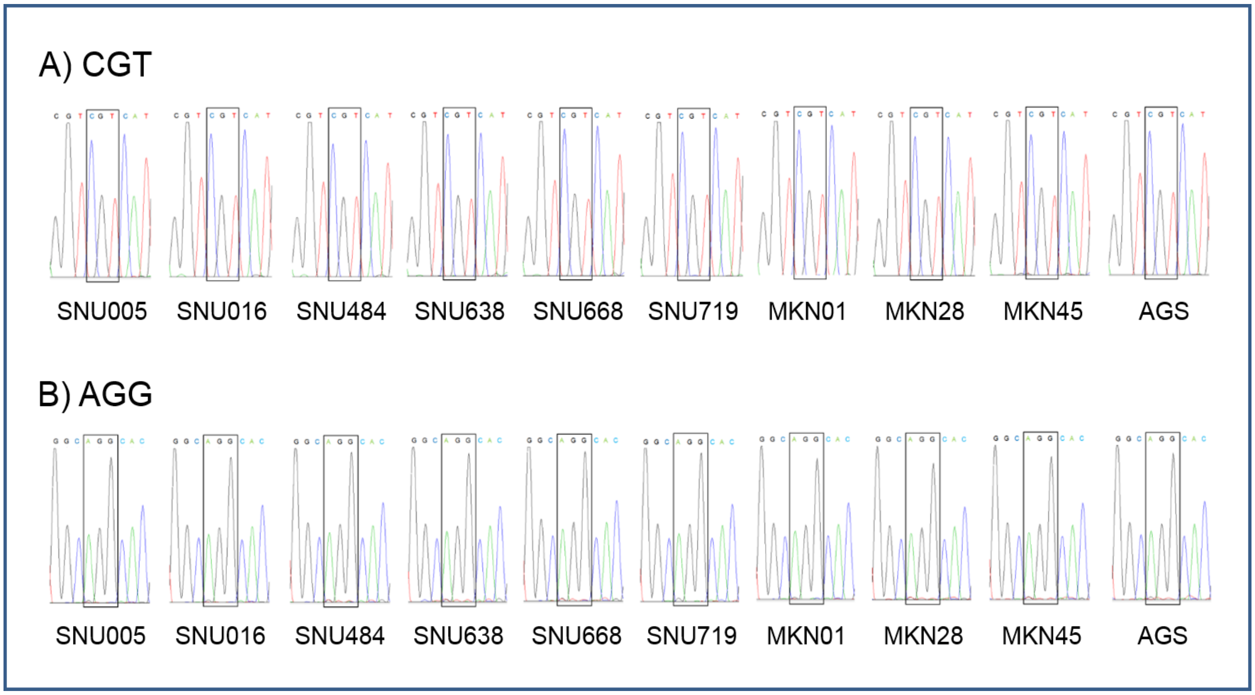

S2 Figure. Sequencing analysis to detect *IDH*1 or *IDH*2 mutations in 10 GC cell lines. (A) *IDH*1 (Arg132), (B) *IDH*2 (Arg172).

S1 Table. Primer sequences used in this study.

Assay	Primer name	Direction*	Sequence	Annealing temp./ number of cycles
Amplification and Sanger sequencing for *IDH*1/2	IDH1^{R132}	F	5′-ACCAAATGGCACCATACGAA-3′	60°C / 35
		R	5′-TGTGTTGAGATGGACGCCTA-3′	
	IDH2^{R172}	F	5′-AATTTTAGGACCCCCGTCGT-3′	60°C / 35
		R	5′-TGTGGCCTTGTACTGCAGAG-3′	
Pyrosequencing of *IDH*1/2	IDH1	F	5′-GTGGCACGGTCTTCAGAGAA-3′	60°C / 37
		R	5′-biotin-TGCCAACATGACTTACTTGATCC-3′	
		S	5′-GGGTAAAACCTATCATCATA-3′	
	IDH2	F	5′-ACATCCCACGCCTAGTCCCT-3′	60°C / 37
		R	5′-biotin-CTCTCCACCCTGGCCTACC-3′	
		S	5′-AAGCCCATCACCATT-3′	
Quantitative real-time RT-PCR	TET1	F	5′-TCTGTTGTTGTGCCTCTGGA-3′	60°C / 40
		R	5′-TTTTGTTCTTCCCCATGACC-3′	
	TET2	F	5′-TTGGACTTCTGTGCTCATGC-3′	60°C / 40
		R	5′-CATCCTCAGGTTTTCCTCCA-3′	
	TET3	F	5′-CCCACAAGGACCAGCATAAC-3′	60°C / 40
		R	5′-CCATCTTGTACAGGGGGAGA-3′	
MBD-seq and RRBS	Adaptors	F	5′-ACACTCTTTCCCTACACGACGCTCTTCCGATCT-3′	
		R	5′-P-GATCGGAAGAGCACACGTCTGAACTCCAGTCAC-3′	
	Amplification	F	5′-AATGATACGGCGACCACCGAGATCTACACTCTTTCCCTACACGACGCTCTTCCGATCT-3′	63°C / 18
		R	5′-CAAGCAGAAGACGGCATACGAGCTCTTCCGATCT-3′	
Bisulfite sequencing	TET1 CGI 5′-shore	F	5′-TTGTGTTATGATTGTTGGAAGAAGTA-3′	59°C / 37
		R	5′-TCCCAAAAAAACTCTAAAAAAAATTT-3′	
	TET1 CGI 3′-shore	F	5′-AGGGAGTAGAGTGTGTTTTGTTAAAAT-3′	58°C / 37
		R	5′-CCAATCCCTTCCAAACCACTTCTTAT-3′	
	TET1 CGI	F	5′-TTTGAGGTTTGTTTTGGGGAGATAT-3′	60°C / 37
		R	5′-CCCCATCCCCACAACTTTA-3′	
Pyrosequencing	TET1 CGI 5′-shore	F	5′-biotin-GGGTTAAATAATTAATAGAAATATTATGA-3′	53°C / 35
		R	5′-TCAAAAATAACACCATTAAACAATATCT-3′	
		S	5′-CACCATTAAACAATATCTAAATC-3′	
	TET1 CGI 3′-shore	F	5′-GAGTTGGTTGGGAGTTGTAAGGATTA-3′	60°C / 35
		R	5′-biotin-ACCAAAACCAAACCCCTCAC-3′	
		S	5′-AGAAATTTATTTATGAGTATTATTG-3′	
	TET1 CGI	F	5′-TTTGAGGTTTGTTTTGGGGAGATAT-3′	60°C / 35
		R	5′-biotin-CCCCATCCCCACAACTTTA-3′	
		S	5′-TGTGGATTTTTGGGAA-3′	
MethyLight	TET CGI 3′-shore	F	5′-TTATTAGTTTTCGTTTGGCGGGATT-3′	59°C / 50
		R	5′-GAAAACCAAACCCCTCACGTACTA-3′	
		Probe	6FAM5′-CGACCAACCCCAACACCCAACTCCG-3′TAMRA	
Luciferase assay	TET1 CGI 3′-shore	F	5′-ATCCTAGGGAGAGTTTTGGGA-3′	60°C / 25
		R	5′-ATACTAGTGACGAGTTGGGGG-3′	
ChIP-PCR	TET1 CGI 5′-shore	F	5′-AGGTCCAGGGCCAAATAACT-3′	60°C / 37
		R	5′-AGAAGGTGCCAGGTCAGAGA-3′	
	TET1 CGI 3′-shore	F	5′-CCTTGCGATCCCATCAGT-3′	63°C / 37
		R	5′-GGGAAGCACAGAGAGAGAGG-3′	
	TET1 CGI	F	5′-GTCTGTCCTGGGGAGACACT-3′	60°C / 37
		R	5′-AGGTAGGGCTGCATGACTTG-3′	

*F, forward; R, reverse; S, sequencing.

S2 Table. Pyrosequencing results for screening *IDH*1/2 mutations in 38 primary GTs.

Sample ID	*IDH*1-1 (CGT -> NGT)				*IDH*1-2 (CGT -> CNT)				*IDH*2-1 (AGG -> NGG)				*IDH*2-2 (AGG -> ANG)			
	A	C	G	T	A	C	G	T	A	C	G	T	A	C	G	T
112T	5.90	**87.90**	6.20	0.00	4.50	0.00	**95.40**	0.10	**96.70**	0.00	1.60	1.70	0.00	0.00	**98.50**	1.50
116T	4.90	**86.70**	5.70	2.70	5.60	0.60	**93.60**	0.20	**96.50**	0.00	1.20	2.30	0.00	0.00	**98.70**	1.30
117T	4.60	**91.00**	4.40	0.00	5.10	0.00	**94.80**	0.10	**91.40**	0.00	7.40	1.20	0.00	0.00	**97.90**	2.10
121T	3.80	**86.70**	6.00	3.50	5.00	0.00	**94.90**	0.10	**92.50**	0.00	7.50	0.00	0.00	0.00	**98.40**	1.60
214T	5.00	**86.20**	5.80	3.00	4.70	0.00	**95.20**	0.10	**98.10**	0.00	0.30	1.60	4.20	0.00	**95.80**	0.00
221T	4.00	**89.60**	2.90	3.50	6.20	0.00	**93.70**	0.10	**91.30**	0.00	8.70	0.00	1.40	0.00	**88.80**	9.80
222T	3.80	**86.50**	7.00	2.70	5.10	0.00	**94.80**	0.10	**96.40**	0.00	2.10	1.50	0.00	0.00	**98.20**	1.80
224T	5.40	**94.50**	0.10	0.00	6.10	0.00	**93.80**	0.10	**92.70**	0.00	6.20	1.10	1.90	0.00	**89.40**	8.70
234T	4.30	**93.50**	2.20	0.00	4.70	0.00	**95.20**	0.10	**95.10**	0.00	3.90	1.00	0.80	0.00	**96.00**	3.20
237T	0.00	**96.00**	4.00	0.00	5.00	0.00	**94.90**	0.10	**93.00**	0.00	5.90	1.10	0.00	0.00	**95.00**	5.00
307T	4.10	**90.60**	5.30	0.00	5.60	0.00	**94.30**	0.10	**98.70**	0.00	0.10	1.20	0.00	0.00	**97.70**	2.30
317T	4.10	**90.70**	5.20	0.00	5.80	0.00	**94.10**	0.10	**98.70**	0.00	0.10	1.20	0.00	0.00	**98.10**	1.90
318T	4.90	**95.00**	0.10	0.00	5.10	0.00	**94.80**	0.10	**98.50**	0.00	0.10	1.40	0.00	0.00	**98.50**	1.50
323T	4.60	**94.40**	1.00	0.00	5.80	0.00	**94.10**	0.10	**97.60**	0.00	1.10	1.30	0.00	0.00	**98.10**	1.90
344T	4.30	**95.60**	0.10	0.00	5.00	0.00	**94.90**	0.10	**98.60**	0.00	0.30	1.10	0.00	0.00	**98.60**	1.40
347T	4.60	**95.30**	0.10	0.00	4.90	0.00	**95.00**	0.10	**93.30**	0.00	5.30	1.40	0.90	0.00	**97.40**	1.70
349T	4.50	**92.10**	3.40	0.00	5.80	0.00	**94.10**	0.10	**94.00**	0.00	4.90	1.10	0.60	0.00	**97.30**	2.10
352T	5.20	**94.70**	0.10	0.00	5.00	0.00	**94.90**	0.10	**98.10**	0.00	0.30	1.60	0.00	0.00	**98.50**	1.50
357T	5.50	**93.70**	0.80	0.00	5.20	0.00	**94.70**	0.10	**99.00**	0.00	1.00	0.00	0.00	0.00	**97.40**	2.60
419T	4.40	**92.40**	3.20	0.00	5.40	0.00	**94.50**	0.10	**89.00**	0.00	10.00	1.00	0.00	0.00	**97.40**	2.60
422T	4.20	**92.50**	3.30	0.00	5.20	0.00	**94.70**	0.10	**100.00**	0.00	0.00	0.00	0.00	0.00	**98.40**	1.60
434T	4.20	**90.00**	5.80	0.00	4.50	0.00	**95.40**	0.10	**91.70**	0.00	7.30	1.00	0.00	0.00	**98.00**	2.00
443T	3.70	**88.30**	8.00	0.00	8.30	0.00	**91.60**	0.10	**93.00**	0.00	4.90	2.10	4.40	0.00	**92.90**	2.70
516T	4.00	**90.60**	5.40	0.00	6.00	0.00	**93.90**	0.10	**94.40**	0.00	4.50	1.10	0.00	0.00	**97.70**	2.30
528T	4.40	**94.00**	1.60	0.00	5.80	0.00	**94.10**	0.10	**94.70**	0.00	3.90	1.40	0.00	0.00	**98.00**	2.00
535T	4.20	**93.00**	2.80	0.00	5.80	0.00	**94.10**	0.10	**98.70**	0.00	0.10	1.20	2.20	0.00	**95.90**	1.90
537T	3.80	**85.50**	7.60	3.10	4.80	0.00	**95.10**	0.10	**95.50**	0.00	3.20	1.30	0.00	0.00	**98.50**	1.50
605T	4.20	**95.40**	0.40	0.00	5.40	0.00	**94.50**	0.10	**98.40**	0.00	0.40	1.20	5.00	0.00	**92.80**	2.20
619T	4.10	**91.40**	4.50	0.00	4.60	0.00	**95.30**	0.10	**93.50**	0.00	6.50	0.00	0.00	0.00	**98.40**	1.60
620T	3.80	**92.70**	3.50	0.00	6.10	0.00	**93.80**	0.10	**95.50**	0.00	3.50	1.00	0.00	0.00	**98.20**	1.80
621T	5.10	**93.70**	0.10	1.10	4.90	0.00	**95.00**	0.10	**93.40**	0.00	5.50	1.10	0.00	0.00	**98.20**	1.80
625T	3.90	**94.30**	1.80	0.00	6.20	0.00	**93.70**	0.10	**96.30**	0.00	2.70	1.00	5.70	0.00	**94.30**	0.00
626T	4.10	**90.10**	5.80	0.00	4.40	0.00	**95.50**	0.10	**100.00**	0.00	0.00	0.00	0.00	0.00	**98.40**	1.60
627T	3.90	**93.40**	2.70	0.00	6.70	0.00	**93.20**	0.10	**98.70**	0.00	0.20	1.10	5.80	0.00	**91.80**	2.40
635T	3.70	**88.50**	7.80	0.00	5.20	0.00	**94.70**	0.10	**91.10**	0.00	7.80	1.10	0.00	0.00	**100.00**	0.00
638T	3.90	**87.80**	8.30	0.00	5.30	0.00	**94.60**	0.10	**96.20**	0.00	2.40	1.40	4.50	0.00	**93.60**	1.90
647T	4.00	**93.10**	2.90	0.00	6.50	0.00	**93.40**	0.10	**94.70**	0.00	3.80	1.50	0.00	0.00	**97.60**	2.40
112N	3.90	**92.70**	3.40	0.00	5.00	0.00	**94.90**	0.10	**90.00**	0.00	8.90	1.10	0.00	0.00	**98.40**	1.60
116N	4.10	**95.80**	0.10	0.00	6.10	0.00	**93.80**	0.10	**96.60**	0.00	2.40	1.00	0.60	0.00	**98.00**	1.40
117N	4.40	**95.50**	0.10	0.00	4.90	0.00	**95.00**	0.10	**93.60**	0.00	5.20	1.20	0.00	0.00	**98.10**	1.90
323N	0.00	**100.00**	0.00	0.00	5.90	0.00	**94.00**	0.10	**94.10**	0.00	5.90	0.00	0.00	0.00	**97.50**	2.50
344N	4.10	**95.60**	0.00	0.30	4.80	0.00	**95.10**	0.10	**98.70**	0.00	0.10	1.20	0.00	0.00	**98.60**	1.40
347N	4.30	**88.10**	7.00	0.60	6.20	0.00	**93.70**	0.10	**93.50**	0.00	6.50	0.00	0.00	0.00	**97.90**	2.10
